# Concussion Symptoms Scale and the Association with Temperature, Equipment, and Play Duration in Non-Concussed Football Players

**DOI:** 10.3390/sports14040133

**Published:** 2026-03-31

**Authors:** Rachel Matthews, Ankur Verma, Derek Calvert, Nathan P. Lemoine, Jack Marucci, Stephen Etheredge, Robert Zura, Guillaume Spielmann, Neil M. Johannsen

**Affiliations:** 1 Department of Orthopaedics, School of Medicine, Louisiana State University Health Sciences Center, New Orleans, LA 70112, USA; 2 Institute for the Health & Performance of Champions, Louisiana State University, Baton Rouge, LA 70802, USA; 3 Department of Orthopaedics, University of Illinois-Chicago, Chicago, IL 60612, USA; 4 LSU Athletics, Louisiana State University, Baton Rouge, LA 70802, USA; 5 School of Kinesiology, Louisiana State University, Baton Rouge, LA 70802, USA; 6 Human Genomics, Pennington Biomedical Research Center, Baton Rouge, LA 70808, USA; 7 Preventive Medicine, Pennington Biomedical Research Center, Baton Rouge, LA 70808, USA

**Keywords:** concussion screening, college football players, symptoms checklist

## Abstract

Background: Symptom scales are routinely used in sport during concussion screening and return-to-play. Limited research has explored the presence of concussion symptoms in the absence of a diagnosed concussion. This study analyzed concussion symptom scores in concussed vs. non-concussed football players after football activities and evaluated the effect of field of play variables. Methods: NCAA Division I football players with (*n* = 9) and without (*n* = 30) diagnosed concussion completed concussion symptom scales (C3 Logix) following practice for 1 week. Wet bulb globe temperature (WBGT), play duration, equipment, and location (inside/outside) were recorded. Mixed models analyzed the effect of day, WBGT, equipment, location, and play duration on concussion-like symptoms in non-concussed players and determined the time course of symptom relief in concussed players. Results: Fatigue or low energy (27.6%), neck pain (16.8%), feeling slowed down (14.8%), and headache (12.8%) were most reported. In non-concussed players, total symptoms scores were higher early in the week (Monday/Tuesday) and decreased throughout the week (*p* < 0.01). No effect of play duration (*p* = 0.49), WBGT (*p* = 0.12), equipment (*p* = 0.40), or location (*p* = 0.83) was found. Symptom scores were greater in the concussed vs. non-concussed groups on days 1–3. Conclusions: Football players report concussion-like symptoms in the absence of a concussion diagnosis, particularly following the first few practices after a game.

## 1. Introduction

Symptom scales have been used as a tool to make informed decisions in sports concussion screening and return-to-play protocols [1,2,3]. Numerous studies have investigated the validity of symptom scales; however, previous literature has centered on assessing athletes after head trauma [4]. Fewer studies have investigated individuals who may report experiencing symptoms generally assessed on the concussion scales in the absence of head trauma [5,6,7].

Current evidence supports the hypothesis that symptom scales identify concussion-like symptoms in the absence of head trauma in healthy individuals. A study of 11,759 individuals from a representative general population sample found that a high proportion of adults aged 18–75 years without a head injury reported post-concussion-like symptoms, including fatigue (41.4%), headaches (35.8%), sleeping problems (35.3%), and being irritable (34.3%) [7]. Similar high rates of concussion-like symptoms were noted in university students, where fatigue (76.9%), longer time to think (60.3%), and poor concentration (58.7%) were the most frequently endorsed items despite no decrements in most of the neuropsychological performance tests [6]. Additionally, 20.3% of adolescent student-athletes reported symptoms consistent with a post-concussion syndrome diagnosis during pre-participation physical examinations, with post-concussion syndrome symptom endorsement being 2.2 times more likely in White compared to Black student-athletes [8]. Prevalence of concussion-like symptoms may range from 35.9% to 75.7% for any experience of the symptoms, and 2.9% to 15.5% for more severe symptoms experienced in the past 2 weeks [9]. Furthermore, summed symptom scores were significantly greater in healthy, physically active individuals immediately following 15 min of high-intensity cycling exercise [10].

A dearth of evidence exists in the medical literature investigating the association of reported symptom scales in a healthy athletic population and ancillary factors that may alter symptom scores in the field of play, specifically temperature, equipment used, and play duration. Factors such as heat, exertion during practice, and competition stressors may be associated with concussion-like symptoms and elevate symptom scale scores to levels that mimic those typically seen in concussions. In the absence of a head hit or whiplash, the presence of concussion-like symptomology may give a false impression of a mild traumatic brain injury and consequently erroneously guide an athlete’s return-to-play course.

Therefore, the present study primarily aimed to examine the reported symptom scores of non-concussed collegiate football players by analyzing fluctuations in symptom scores during a week of football activity. Furthermore, our secondary aim was to investigate potential relationships between symptom scores and variables associated with the field of play, including environmental temperature, equipment used, and play duration, in non-concussed, collegiate football players. Lastly, we aim to determine the time course of relief of concussion symptoms in concussed vs. non-concussed players. These aims provide a novel contribution to the existing literature by employing a repeated measures design in a non-concussed population to analyze fluctuations across a week, investigating variables which may influence concussion-like symptom endorsement, and specifically focusing on football, a contact sport where concussion screening and treatment are particularly important.

## 2. Materials and Methods

### 2.1. Participants

Participant anthropometric data are summarized in Table 1. Participants were recruited from a National Collegiate Athletic Association (NCAA) Division I college football team by the team’s athletic training department in accordance with the study’s inclusion and exclusion criteria proposed below. A total of 30 participants between the ages of 18 and 23 met the inclusion criteria and volunteered to participate in the study. An attempt was made to have an equally representative sample across football positions. The non-concussed cohort included 10 linemen (defensive and offensive line), 19 skills players (defensive back, linebacker, running back, and wide receiver), and 1 special teams player (kicker). The study was approved by the Louisiana State University Institutional Review Board (IRB# 4109), and informed consent was obtained from each participant prior to any assessments. Inclusion criteria included American Football players who were on a NCAA Division I roster during a single season. The players had to be willing to undergo a concussion symptoms score checklist questionnaire for one week after practice and games, capable and willing to give written informed consent, and understand the exclusion criteria. In addition, only players who actively participated during the week were recruited. All players were cleared by team physicians to participate in the activity. Players were excluded from the study if they had any conditions the team physician regarded as being too risky to participate in athletics, had been diagnosed with a concussion by a team physician during the previous year of their enrollment, or did not practice or had limited activity during the week. Additionally, volunteers were excluded for any other medical, psychiatric, or behavioral factors that, in the judgment of the Principal Investigator, may have interfered with study participation or the ability to follow the protocol. The investigator of this study had access to data collected under the supervision of the team physician to determine final eligibility.

In addition, data were compiled on 9 past players with a diagnosed concussion to determine the normal time-course for changes in subjective symptomology as well as the variability in response. Data were retrospectively analyzed from players who sustained concussions during the same year as data collection for the non-concussed group. Due to the diagnosed concussions, these players were restricted or limited in their participation in games and practice; therefore, questionnaires were not collected after physical activity like the non-concussed group. Participant characteristics are included in Table 1. Participants included 3 linemen (defensive and offensive line), 5 skills players (defensive back, tight end, wide receiver), and 1 special teams player (long snapper).

Power analysis was not conducted for the concussed group since data were limited to the number of players who sustained concussions in a single season. Additionally, power analysis was not conducted for the non-concussed group due to expected limitations in recruitment.

### 2.2. Study Design

The present study was an observational, prospective study of concussion symptom scores in Division I collegiate American Football players. During 6 weeks of an NCAA football season, 5 different Division I college football players were recruited on a weekly basis. The athletes were administered a graded concussion symptoms checklist questionnaire (C3 Logix, Cleveland, OH, USA) post-activity for one week (constituted Monday through Sunday). Practices were held every day except for Saturday, which was reserved for games, and Sunday, which was an off day used for recovery. The graded concussion symptoms checklist was the same one used for suspected concussions and was administered by a certified athletic trainer for the football team. The symptoms checklist was administered to the selected 5 players each day during the week immediately after practices, games, and off-days. At the start of a new week, 5 new athletes were recruited and consented, and the process was repeated.

### 2.3. Study Assessments

The C3 Logix graded symptom checklist was administered to players on an iPad every day for 1 week and consisted of 27 symptoms, including headache, dizziness, difficulty concentrating, and confusion. Each symptom was reported on a scale of 0–6 (0 = none, 1 or 2 = mild, 3 or 4 = moderate, 5 or 6 = severe), and the sum of the scores for all symptoms was reported as the total symptom score, with a maximum total symptom score of 162. The rationale and validity of the C3 Logix have previously been described in detail [11,12].

In addition to the concussion symptoms checklist, data for ancillary measures were collected for each day of practice and included average wet bulb globe temperature (WBGT; Kestrel 5400 Heat Stress Tracker, Boothwyn, PA, USA) measured every hour during activity, categories for practice equipment (jerseys only, full pads, shells (shoulder pads and helmets only), and helmets and jerseys only), location (i.e., inside vs. outside), and play duration measured in minutes (Table 2). Additionally, collision data, collected from sensors in the players’ helmets (Head Health Network, Baton Rouge, LA, USA), were used to ensure no participants acquired helmet *g* forces sufficient to affect concussion symptoms (i.e., a “red” hit = 100 g).

### 2.4. Statistical Analysis

All statistical analyses were conducted using JMP Pro 18.0.1 (SAS, Inc., Cary, NC, USA). Each non-concussed player participated for 1 week, from Monday to Sunday (Day 1 to Day 7), and all players’ data were combined by day of the week. Mixed models were used to determine the individual and total concussion symptoms responses (dependent variable; ordinal data) across days of the week (time effect; repeated measure/fixed effect) after accounting for individual participant variability (random effect). Models were also run to determine the effect of WBGT, equipment, location, and play duration (fixed effects) on total concussion symptom scores (dependent variable) in non-concussed players, with participant ID included as a random effect. Last, a 2-way mixed model was run to examine the time-course (time effect; fixed effect) for the alleviation of concussion symptoms (dependent variable) in concussed compared to non-concussed players (nested group; fixed effect) after accounting for individual participant variability (random effect). In the concussed group, Day 1 is the first day after concussion diagnosis. Where a time*concussion interaction was observed, post hoc analyses compared total symptom scores from individual days in the concussed group to total scores for all days in the non-concussed group to establish consistent alleviation of symptoms, despite potential fluctuations in daily symptom endorsement. Data are presented as mean ± standard deviation unless otherwise specified and significance is set at *p* < 0.05. Where appropriate, post hoc analyses were conducted using Student’s *t*-test.

## 3. Results

Non-concussed players completed the symptom score questionnaire with 93.3% compliance. Across the 7 days, total concussion symptoms scores were 2.8 ± 3.5 (overall mean ± within-subject standard deviation) for non-concussed players and ranged from 0 to 32. A summary of symptom endorsement for the non-concussed players is presented in Table 3. Fatigue or low energy (27.6% of completed concussion symptom checklist questionnaires), neck pain (16.8%), feeling slowed down (14.8%), and headache (12.8%) are among the most commonly endorsed symptoms, while blurred vision (0.0%), ringing in the ears (0.0%), dizziness (0.5%), and sensitivity to noise (0.5%) are the least common. Neck pain was the only symptom with “severe” endorsement and occurred in 0.5% of questionnaires.

**Figure 1:**
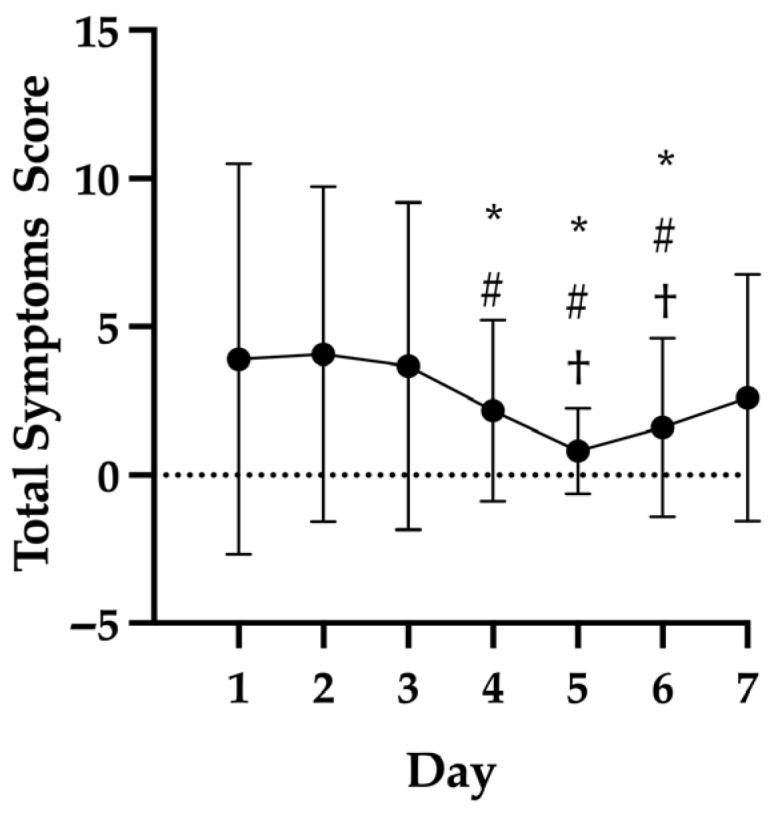
Total concussion symptoms scores across the week in non-concussed football players. Day 1 represents Monday. * significantly different from Day 1, *p* < 0.05. # significantly different from Day 2, *p* < 0.05. † significantly different from Day 3, *p* < 0.05. Data are mean ± standard deviation.

**Figure 2:**
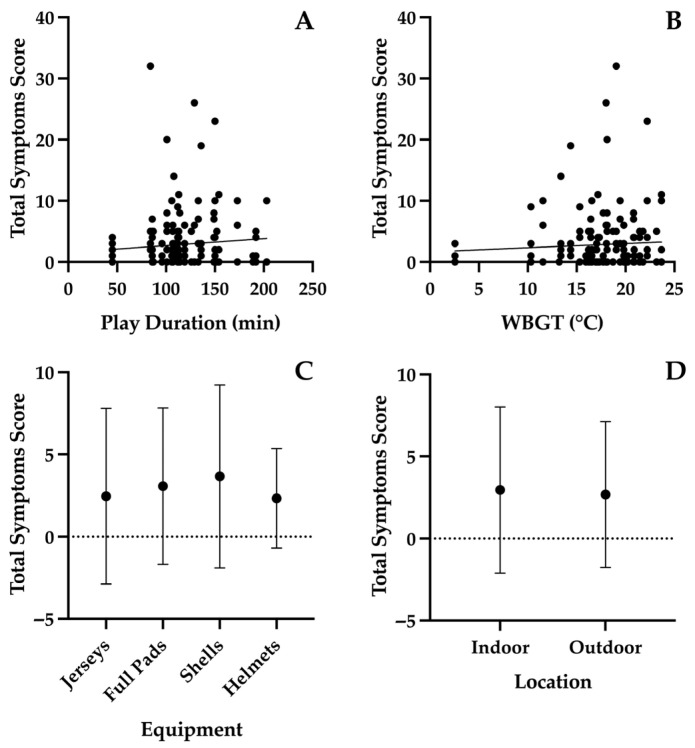
Effect of play duration (**A**), wet bulb globe temperature (WBGT; (**B**)), equipment (**C**), and location (**D**) on total concussion symptoms scores in non-concussed players. Data points in figures (**A**,**B**) represent total scores for each completed concussion symptoms questionnaire. Data in figures (**C**,**D**) are mean ± standard deviation.

In non-concussed players, total symptom scores were higher at the start of the week and decreased as the week progressed (*p* < 0.01; Figure 1); specifically, symptoms scores were higher on Monday (mean ± 95% CI; 3.9 ± 2.5) and Tuesday (4.1 ± 2.1) compared to Thursday through Saturday (1.6 ± 0.6) and greater on Wednesday (3.7 ± 2.1) than Friday (0.8 ± 0.6) and Saturday (1.6 ± 1.0). Individual symptom scores did not change across the week (*p* > 0.05), with the exception of fatigue or low energy (*p* < 0.01), trouble falling asleep (*p* = 0.02), nervous or anxious (*p* < 0.01), and sleeping less than usual (*p* = 0.01), which were all greatest on Monday or Tuesday. No effect of play duration (*p* = 0.49; Figure 2A), WBGT (*p* = 0.12; Figure 2B), equipment (*p* = 0.40; Figure 2C), or location (*p* = 0.83; Figure 2D) was found on total symptom scores. However, the WBGT was lower and less variable (17.6 ± 4.2 °C; Table 2) over the study period than anticipated.

**Figure 3:**
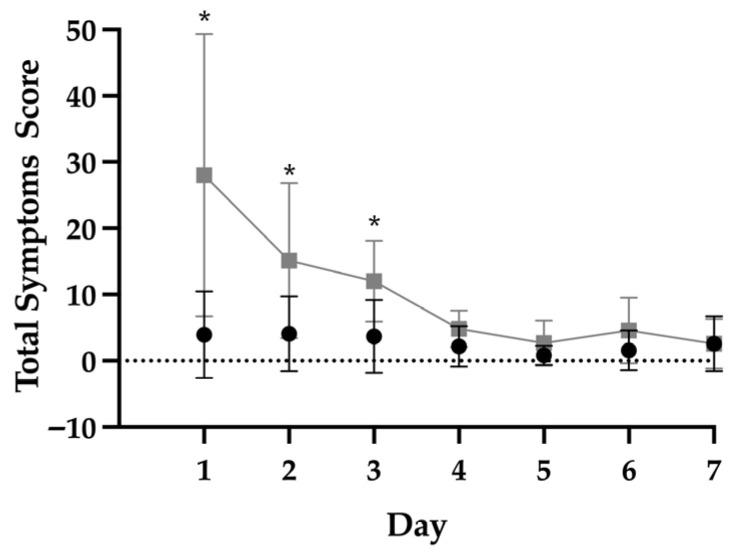
Total concussion symptoms scores across the week in concussed (■) vs. non-concussed (●) football players. In non-concussed players, day 1 represents Monday. * significant difference between concussed players on a specific day and non-concussed players on all days, *p* < 0.05. Data are mean ± standard deviation.

Individual symptom endorsement for the concussed group can be found in Table S1 of the Supplementary Materials. An effect of time, concussion group (i.e., concussed vs. non-concussed), and interaction was found (*p* < 0.01 for all; Figure 3), whereby total symptoms scores were greater in the concussed players on days 1 through 3 compared to all days in the non-concussed players. Additionally, total scores were greater on day 4 in the concussed group compared to days 5 and 6 for the non-concussed group. The effects of the concussion group and interaction found on total symptom scores were similar when analyzing specific symptoms and are reported in Table S2 of the Supplementary Materials.

## 4. Discussion

The present study aimed to examine fluctuations in concussion-like symptoms across the week in non-concussed, collegiate football players and investigate potential field-of-play-related modulators of symptom scores. As hypothesized, symptom scores were greater at the start of the week and decreased as the week progressed; however, symptom scores were not elevated following game day. Furthermore, play duration, WBGT, equipment, and location had no effect on symptom scores. Lastly, symptom scores in the concussed players were not different from the non-concussed group 4 days after injury.

Significant elevations in symptom scores were found at the start of the week compared to the end of the week, possibly due to lingering effects of game day, where the combination of mental and physical exertion is greater compared to practice. Surprisingly, concussion symptom scores were not greater immediately after the game on Saturday or during the rest day on Sunday. Although not measured in the present study, competition-induced stress may mitigate pain sensitivity and reports of discomfort [13], thereby lowering the presence of positive symptom scores following game play whilst elevating subsequent concussion symptom scores when exposed to physical stress in the days after. Interestingly, the prevalence of concussion symptoms appeared to be lower in the present population compared to previously reported data in healthy adults and college students [6,7]. Previous research has demonstrated that collegiate football players have a strong understanding of the signs and symptoms of concussions, but may not report concussion symptoms due to motivation to continue playing from the identity of being a football player and fear of letting the team down [14]. Consequently, players in the present study may have been apprehensive about reporting concussion-like symptoms for fear that it may affect their ability to play.

No effect of WBGT, location, or equipment on symptom score was identified, possibly due to the timing of the study, which was started in October and finished in November. Given that the college is in the south, it is plausible that the players were acclimatized to playing in hot and humid conditions, even with the addition of helmets and pads, and were not exposed to sufficient thermal stress to trigger concussion-like symptomology during the study period. If the study was conducted during pre-season training camp, where environmental conditions are significantly hotter and more humid, WBGT and equipment may have had a greater impact on symptom scores. Several similarities exist between heat-related illness and concussion symptoms, including headache, dizziness, and fatigue [15,16], which may not be differentiated between with a questionnaire, such as the C3 Logix used in the present study, and elevate the concussion symptoms scores reported. Consequently, additional considerations are required when interpreting symptom scores to minimize the risk of false positives or false negatives in diagnosing concussions.

Furthermore, no effects of play duration on symptom scores were found. Previous research has found that symptom scores are elevated following exercise in non-concussed children and adolescents [17]. Additionally, high-intensity, but not moderate-intensity, exercise significantly increases symptom scores immediately post-exercise [10]. Therefore, it is possible that exercise intensity, player load, and impact load are more relevant to the reporting of positive concussion symptom scores than play duration alone. Future research should consider utilizing wearable technology, accelerometers, or heart rate monitors to quantify internal and external training load, investigate the effect on concussion-like symptoms, and control for the effect of inter-week variability in training load across all participants.

When comparing the concussed and non-concussed populations, the greatest difference was observed between the start of the week and the start of the concussion protocol. The majority of concussed players saw the greatest decrements in symptom score within the first 3 days, and symptom scores were no longer different at day four compared to the non-concussed group on any day; however, one player experienced sustained elevations in symptom scores at 11 days post-injury. Previous research supports the proposed time course for symptom recovery and suggests that self-reported symptoms recover in 6–9 days in collegiate athletes [18], although these results do not appear to translate to younger, high school athletes [19].

Although the present study successfully recruited a large sample size of D1 collegiate football players (N = 39) to participate in a longitudinal study with high compliance (93%), relative limitations should be considered when interpreting the data. As previously discussed, WBGT was lower and less variable than anticipated; therefore, future research may benefit from collecting measurements across a whole year or from multiple sites to capture a wider range of thermal stress. Data collection from multiple sites would have also allowed for a larger sample size. Additionally, symptom scales are inherently subjective in nature and may be influenced by mental or emotional state, physical stress, or physiological challenges associated with being a collegiate athlete [20,21]. Consequently, a limitation of the present study was a lack of objective testing measures such as the Balance Error Scoring System (BESS) test, physical exam, or computerized reaction time test. Incorporating objective measures may have provided a more comprehensive understanding of how external factors may contribute to overall concussion management and identify if daily fluctuations in self-reported symptoms translate to concussion-like impairments in physical or neuromuscular function. Furthermore, due to the nature of the sport and the requirements for game travel, the location of data collection sometimes varied. Previous research has demonstrated that the testing environment may influence BESS test outcomes [22,23], and although the present study used a self-reported rather than an objective measure, it is possible that the competitive environment, game outcome, quality of light, and noise also influenced the reported outcomes. While all efforts were utilized to ensure that our testing population had no actual concussions, a possibility of not reporting or underreporting concussions always exists.

In conclusion, athletes may report positive concussion-like symptom scores in the absence of a diagnosis of concussion. Using data collected from a small sample from a single team, the presence of concussion-like symptoms may be greater at the start of the week, following the first few practices after game day. Consequently, multiple baseline tests may provide a better insight into an individual’s normal range for symptom reporting and establish personalized recovery in the case of a player sustaining a concussion. The present study did not identify significant correlations between symptom scores and temperature or play duration, possibly due to acclimatization, a lack of exposure to thermal stress during the study period, and a greater effect of exercise intensity than duration of symptom reporting. In accordance with other studies, data suggest that symptom scores return to baseline approximately 4 days post-concussion, whereby symptom scales alone may be unable to differentiate between normal concussion-like symptoms and lingering effects of a sustained concussion. Additional objective measures of concussion management, including BESS and reaction time tests, may provide useful insight into the interplay between variability in objective and subjective measures in concussed and non-concussed players.

## Figures and Tables

**Table 1:** Participant characteristics.

	Non-Concussed Group (n = 30)		Concussed Group (n = 9)	
	Mean ± SD	[min, max]	Mean ± SD	[min, max]
Age (y)	20.3 ± 1.6	[18, 23]	20.2 ± 1.8	[18, 23]
Height (m)	1.89 ± 0.06	[1.75, 2.01]	1.91 ± 0.06	[1.80, 1.96]
Weight (kg)	110.4 ± 23.0	[82.6, 152.0]	107.7 ± 18.0	[83.5, 138.3]
BMI (kg/m^2^)	30.8 ± 5.2	[23.8, 40.8]	29.4 ± 3.8	[23.0, 36.2]

**Table 2:** Summary of ancillary measures collected on each active day in non-concussed players.

	Day						Total
	1	2	3	4	5	6	
Equipment	Jerseys	Full Pads	Shells	Helmets	Jerseys	Full Pads	
Location (n/N)							
Inside	6/6	2/6	3/6	6/6	6/6	0/6	23/36
Outside	0/6	4/6	3/6	0/6	0/6	6/6	13/36
Duration (min)	99.3 ± 24.6	134.7 ± 19.0	119.2 ± 10.7	101.7 ± 26.1	56.6 ± 21.9	151.4 ± 77.9	111.8 ± 45.1
WBGT (°C)	19.2 ± 1.8	18.3 ± 4.7	17.9 ± 3.1	17.5 ± 1.2	14.1 ± 6.7	18.2 ± 4.9	17.6 ± 4.2

WBGT: wet bulb globe temperature. For location (n/N), data reflects the number of practices or games held inside/outside (n) out of the total number of practices/games (N). For duration and WBGT, data are presented as mean ± standard deviation.

**Table 3:** Summary of symptom endorsement in non-concussed players.

Symptom	Score							Total Positive Scores
	None	Mild		Moderate		Severe		
	0	1	2	3	4	5	6	
Fatigue or Low Energy	72.4%	9.7%	9.7%	6.1%	2.0%			27.6%
Neck Pain	83.2%	5.6%	4.1%	4.6%	2.0%	0.5%		16.8%
Feeling Slowed Down	85.2%	7.7%	5.1%	1.5%	0.5%			14.8%
Headache	87.2%	8.2%	4.6%					12.8%
Sleeping Less Than Usual	87.8%	5.1%	5.1%	2.0%				12.2%
Pressure in Head	90.3%	6.1%	3.1%	0.5%				9.7%
Trouble Falling Asleep	90.8%	5.6%	2.0%	1.5%				9.2%
Difficulty Sleeping Soundly	90.8%	6.1%	2.0%	1.0%				9.2%
Feeling Like in a Fog	93.4%	5.1%	1.0%	0.5%				6.6%
Don’t Feel Right	93.9%	4.6%	1.0%	0.5%				6.1%
Drowsiness	94.9%	1.5%	2.6%	0.5%	0.5%			5.1%
Difficulty Remembering	94.9%	3.1%	2.0%					5.1%
Irritability	95.4%	2.0%	2.6%					4.6%
More Emotional	95.9%	0.5%	3.1%		0.5%			4.1%
Nervous or Anxious	96.4%	2.6%	1.0%					3.6%
Balance Problems	96.4%	3.6%						3.6%
Sleeping More Than Usual	96.9%	2.6%		0.5%				3.1%
Difficulty Concentrating	97.4%	2.0%	0.5%					2.6%
Sadness	98.0%	0.5%	1.0%	0.5%				2.0%
Nausea or Vomiting	98.0%	1.5%	0.5%					2.0%
Sensitivity to Light	98.5%	1.5%						1.5%
Numbness or Tingling	99.0%	0.5%	0.5%					1.0%
Confusion	99.0%	1.0%						1.0%
Sensitivity to Noise	99.5%		0.5%					0.5%
Dizziness	99.5%	0.5%						0.5%
Ringing in the Ears	100.0%							0.0%
Blurred Vision	100.0%							0.0%

Data are reported as the percentage of completed concussion symptom questionnaires.

## Data Availability

The data presented in this study are available on request from the corresponding author due to ethical reasons.

## Supplementary Materials

The following supporting information can be downloaded at https://www.mdpi.com/article/10.3390/sports14040133/s1, Table S1: Summary of Symptom Endorsement in Concussed Players; Table S2: Descriptive Statistics for Individual Symptoms in Concussed and Non-Concussed Players and Results of 2-Way Mixed Model.

## Abbreviations

The following abbreviations are used in this manuscript:

NCAA	National Collegiate Athletic Association
WBGT	Wet Bulb Globe Temperature
BESS	Balance Error Scoring System
