# Supplementary material for: Concussion Symptoms Scale and the Association with Temperature, Equipment, and Play Duration in Non-Concussed Football Players
Source: Sports (Basel). 2026 Mar 31;14(4):133. doi: 10.3390/sports14040133 (PMC13119781; doi:10.3390/sports14040133)
Supplement: Supplementary file 1 [file sports-14-00133-s001.zip › Supplementary Materials/Table S1.pdf]

Table S1. Summary of Symptom Endorsement in Concussed Players.

Symptom	Score							Total Positive Scores
	None	Mild		Moderate		Severe		
	0	1	2	3	4	5	6	
Headache	31.5%	27.8%	16.7%	11.1%	11.1%	1.9%		68.5%
Pressure in Head	42.6%	25.9%	20.4%	7.4%	3.7%			57.4%
Sensitivity to Light	55.6%	29.6%	7.4%	3.7%	3.7%			44.4%
Dizziness	63.0%	22.2%	7.4%	5.6%	1.9%			37.0%
Don't Feel Right	64.8%	20.4%	9.3%	3.7%	1.9%			35.2%
Fatigue or Low Energy	70.4%	16.7%	9.3%	1.9%	1.9%			29.6%
Neck Pain	72.2%	20.4%	7.4%					27.8%
Sensitivity to Noise	74.1%	13.0%	7.4%	3.7%	1.9%			25.9%
Feeling Slowed Down	74.1%	18.5%	1.9%	3.7%	1.9%			25.9%
Drowsiness	75.9%	14.8%	1.9%	7.4%				24.1%
Blurred Vision	75.9%	18.5%	1.9%	3.7%				24.1%
Sleeping More Than Usual	77.8%	13.0%	7.4%	1.9%				22.2%
Trouble Falling Asleep	79.6%	5.6%	7.4%	7.4%				20.4%
Balance Problems	79.6%	13.0%	1.9%	5.6%				20.4%
Difficulty Concentrating	79.6%	13.0%	5.6%	1.9%				20.4%
Feeling Like in a Fog	79.6%	13.0%	5.6%	1.9%				20.4%
Difficulty Remembering	81.5%	7.4%	7.4%	3.7%				18.5%
Sleeping Less Than Usual	83.3%	5.6%	5.6%	3.7%	1.9%			16.7%
Confusion	83.3%	13.0%	1.9%		1.9%			16.7%
Ringing in the Ears	85.2%	11.1%	1.9%	1.9%				14.8%
Difficulty Sleeping Soundly	87.0%	3.7%	7.4%	1.9%				13.0%
More Emotional	90.7%	1.9%	1.9%	3.7%			1.9%	9.3%
Nausea or Vomiting	90.7%	7.4%		1.9%				9.3%
Sadness	92.6%	3.7%	3.7%					7.4%
Nervous or Anxious	94.4%	1.9%	1.9%	1.9%				5.6%
Irritability	94.4%	3.7%	1.9%					5.6%
Numbness or Tingling	96.3%	1.9%		1.9%				3.7%

Data are reported as the percentage of completed concussion symptom questionnaires.
