# Supplementary material for: Concussion Symptoms Scale and the Association with Temperature, Equipment, and Play Duration in Non-Concussed Football Players
Source: Sports (Basel). 2026 Mar 31;14(4):133. doi: 10.3390/sports14040133 (PMC13119781; doi:10.3390/sports14040133)
Supplement: Supplementary file 1 [file sports-14-00133-s001.zip › Supplementary Materials/Table S2.pdf]

Table S2. Descriptive Statistics for Individual Symptoms in Concussed and Non-Concussed Players and Results of 2-Way Mixed Model.

Symptom	Concussed	Non-Concussed	Effect of Concussion (P-value)	Interaction Effect (P-value)
Headache	1.5 ± 1.4 [1 ± 2]	0.2 ± 0.5 [0 ± 0]	< 0.01 *	< 0.01 *
Pressure in Head	1.0 ± 1.1 [1 ± 2]	0.1 ± 0.5 [0 ± 0]	< 0.01 *	< 0.01 *
Neck Pain	0.4 ± 0.6 [0 ± 1]	0.4 ± 1.0 [0 ± 0]	0.83	0.26
Nausea or Vomiting	0.1 ± 0.5 [0 ± 0]	0.0 ± 0.2 [0 ± 0]	0.04 *	0.17
Dizziness	0.6 ± 1.0 [0 ± 1]	0.0 ± 0.1 [0 ± 0]	< 0.01 *	< 0.01 *
Blurred Vision	0.3 ± 0.7 [0 ± 0]	0.0 ± 0.0 [0 ± 0]	< 0.01 *	< 0.01 *
Balance Problems	0.3 ± 0.8 [0 ± 0]	0.0 ± 0.2 [0 ± 0]	< 0.01 *	< 0.01 *
Sensitivity to Light	0.7 ± 1.0 [0 ± 1]	0.0 ± 0.1 [0 ± 0]	< 0.01 *	< 0.01 *
Sensitivity to Noise	0.5 ± 0.9 [0 ± 1]	0.0 ± 0.1 [0 ± 0]	< 0.01 *	< 0.01 *
Feeling Slowed Down	0.4 ± 0.9 [0 ± 1]	0.2 ± 0.7 [0 ± 0]	0.30	0.01 *
Feeling Like in a Fog	0.3 ± 0.7 [0 ± 0]	0.1 ± 0.4 [0 ± 0]	0.11	< 0.01 *
Don't Feel Right	0.6 ± 0.9 [0 ± 1]	0.1 ± 0.4 [0 ± 0]	< 0.01 *	< 0.01 *
Difficulty Concentrating	0.3 ± 0.8 [0 ± 0]	0.0 ± 0.2 [0 ± 0]	< 0.01 *	< 0.01 *
Difficulty Remembering	0.3 ± 0.8 [0 ± 0]	0.1 ± 0.3 [0 ± 0]	0.02 *	< 0.01 *
Fatigue or Low Energy	0.5 ± 0.9 [0 ± 1]	0.6 ± 1.0 [0 ± 1]	0.83	0.31
Confusion	0.2 ± 0.7 [0 ± 0]	0.0 ± 0.1 [0 ± 0]	< 0.01 *	< 0.01 *
Drowsiness	0.4 ± 0.9 [0 ± 0]	0.1 ± 0.5 [0 ± 0]	0.03 *	0.11
Trouble Falling Asleep	0.4 ± 0.9 [0 ± 0]	0.1 ± 0.5 [0 ± 0]	0.05	0.32
More Emotional	0.3 ± 1.0 [0 ± 0]	0.1 ± 0.5 [0 ± 0]	0.16	0.3 *
Irritability	0.1 ± 0.3 [0 ± 0]	0.1 ± 0.3 [0 ± 0]	0.85	< 0.01 *
Sadness	0.1 ± 0.4 [0 ± 0]	0.0 ± 0.3 [0 ± 0]	0.30	0.63
Nervous or Anxious	0.1 ± 0.5 [0 ± 0]	0.0 ± 0.3 [0 ± 0]	0.41	0.11
Sleeping More Than Usual	0.3 ± 0.7 [0 ± 0]	0.0 ± 0.3 [0 ± 0]	< 0.01 *	0.49
Sleeping Less Than Usual	0.4 ± 0.9 [0 ± 0]	0.2 ± 0.6 [0 ± 0]	0.40	0.40
Difficulty Sleeping Soundly	0.2 ± 0.7 [0 ± 0]	0.1 ± 0.5 [0 ± 0]	0.47	0.05
Ringing in Ears	0.2 ± 0.6 [0 ± 0]	0.0 ± 0.0 [0 ± 0]	< 0.01 *	< 0.01 *
Numbness or Tingling	0.1 ± 0.4 [0 ± 0]	0.0 ± 0.2 [0 ± 0]	0.19	< 0.01 *

Data are presented as mean ± standard deviation [median ± interquartile range]. * significant effect of concussion or concussion*time interaction, $P < 0.05$.
